# Likelihood of obtaining a usable embryo for transfer after IVF with PGT-A and PGT-M for variants in two genes

**DOI:** 10.1007/s10815-026-03868-4

**Published:** 2026-04-24

**Authors:** Melody A. Rasouli, Lisette Collins, Mabel Lee, Rachel Martel, Mehrnaz Siavoshi, Lorna Kwan, Jenna Miller, Chelsea Woody, Robert Roman, Lindsay Kroener

**Affiliations:** 1 Department of Obstetrics and Gynecology, University of California, Los Angeles, Los Angeles, United States; 2 Department of Urology, University of California, Los Angeles, Los Angeles, United States; 3 Cooper Genomics, Livingston, United States

**Keywords:** Preimplantation genetic testing, PGT-M, PGT-A, In vitro fertilization, Monogenic disorders

## Abstract

**Purpose:**

To determine the likelihood of obtaining at least one transferable embryo per cycle in patients undergoing IVF with PGT-A + PGT-M for variants in one versus two genes.

**Methods:**

IVF cycles in patients 18–45 years undergoing PGT-A + PGT-M for variants in one or two genes at a single genetics laboratory were analyzed from 11/2019 to 3/2023. Cycles were stratified by SART age category and disease inheritance pattern. Analyses were performed using median and chi-square tests.

**Results:**

A total of 4634 IVF cycles were included: 146 cycles (3.15%) using PGT-A + PGT-M for two genes and 4488 cycles (96.85%) for one gene. After adjusting for the number of embryos biopsied, the likelihood of having at least one usable embryo was significantly lower in patients testing for variants in two genes compared to one in patients aged < 35 (75.0% v 82.5%, *p* = 0.004) and 35–37 (55.0% v 74.4%, *p* = 0.025) with no difference in other age groups 38–40 (66.7% v 74.4%, *p* = 0.586) and 41–42 (42.9% v 51.7%, *p* = 0.113).

**Conclusions:**

There were significantly lower odds of obtaining a usable embryo when testing for variants in genes in patients aged < 35 and 35–37. No significant difference was seen among patients aged 38–40 or 41–42. These findings provide quantifiable data to counsel patients considering PGT-M testing for variants in two genes.

**Supplementary Information:**

The online version contains supplementary material available at 10.1007/s10815-026-03868-4.

## Introduction

Preimplantation genetic testing for monogenic disorders (PGT-M) has enabled the selection and transfer of embryos that are unaffected by specific single-gene conditions. This technology gives patients with known genetic risks the ability to significantly reduce the likelihood of transmission of inherited disorders to their offspring [1]. By identifying embryos that do not carry the variants associated with these disorders, PGT-M allows for exclusion of genetic conditions in offspring that may be lethal or significantly life-altering.

Carrier screening should be offered to all individuals who are pregnant or planning a pregnancy, either through a traditional targeted approach or an expanded panel [2]. The American College of Obstetricians and Gynecologists recommends screening focused primarily on spinal muscular atrophy (SMA), cystic fibrosis (CF), fragile X syndrome, and certain conditions based on a patient’s ethnicity or family history [3]. However, advances in next-generation sequencing technologies have made expanded carrier screening (ECS) panels more accessible and cost-effective such that the American College of Medical Genetics recommends ECS for all patients [4]. Many ECS panels now routinely screen for up to 500 + genes, encompassing a wide range of autosomal recessive and X-linked conditions. ECS may be offered to all patients, and included conditions should be of significant clinical impact, have a well-defined phenotype, and be associated with early onset and substantial morbidity or mortality [5].

In addition to broader use of ECS, there has also been an increase in genetic testing for other purposes, such as inherited cancer risks. The rapid advancements in genetic testing technologies, coupled with the increased accessibility of genetic carrier screening, have led to a significant rise in the detection of multiple pathogenic variants of genes in individuals and couples seeking reproduction [6]. As a result, a growing number of patients carry more than one pathogenic variant. This increases the complexity of their reproductive decision-making, with the option to undergo PGT-M for more than one gene. Depending on the inheritance pattern of the gene(s), testing may significantly reduce the odds of having a usable embryo.

A number of studies have addressed success rates for IVF alone compared with IVF with PGT-M for a single gene. However, there is little data addressing use of PGT-M for more than one monogenic gene. A single-center case series investigated the indications, trends, and outcomes for PGT-M when performed for two or more monogenic disorders and found a 43% increased use of PGT-M for two or more genes between 2010 and 2021 [7]. Notably, this study was limited by the inclusion of a single site and a very small sample size of 23 patients. As patients present more frequently as carriers of multiple genetic conditions, clinicians face growing challenges in counseling around the probability of success.

Our study aims to address this gap by evaluating IVF cycles in which patients underwent concurrent PGT-A and PGT-M for variants in one and two genes and comparing the likelihood of obtaining at least one euploid, unaffected embryo between the two groups. We anticipated an increased impact of aneuploidy and a lower number of usable embryos based on PGT-A results alone in older patients, with less possible embryos for exclusion based on PGT-M results. In contrast, for younger patients, we expected a higher euploidy rate and more available embryos for PGT-M for two genes and a greater pool of embryos for possible exclusion based on PGT-M. It is possible that the relative impact of PGT-M for two genes will be attenuated by higher aneuploidy rates in older patients. Our study aimed to analyze these outcomes and to assess how the presence of multiple genes impacts the probability of obtaining an embryo for transfer. These findings provide valuable insights into the success rates and challenges associated with PGT-M for two genes, thereby informing clinical practice and enhancing patient counseling. We hypothesized that concurrent testing for two genes would reduce the likelihood of obtaining a usable embryo compared with testing for one gene, particularly in younger patients.

## Methods

All IVF cycles from patients aged 18–45 undergoing PGT-A with concurrent PGT-M for one or two genes at a single genetics laboratory from November 2019 through March 2023 were analyzed. The study was submitted for Institutional Review Board (IRB) approval and was found to be exempt by the University of California Los Angeles IRB. Cycles were stratified by SART age categories: < 35 years, 35–37 years, 38–40 years, 41–42 years, and > 42 years. PGT-M cycles were stratified by disease inheritance pattern: autosomal recessive (AR), autosomal dominant (AD), and X-linked (XL). X-linked recessive and X-linked dominant conditions were combined due to small numbers. Exclusion criteria included cycles using oocyte donors, monogenic disorders without a clear inheritance pattern, embryos that underwent PGT-M but not PGT-A testing, and biopsies that failed to yield results.

As outlined in our previous publication [8], all trophectoderm biopsies underwent next-generation sequencing (NGS) for PGT-A and/or PGT-M by CooperSurgical, Inc. (Livingston, NJ). The trophectoderm biopsies were lysed and amplified using the SurePlexTM DNA Amplification System (November 2019 to February 2023) per manufacturer’s instructions (Illumina, Inc., San Diego, CA) or Primary Template-Directed Amplification (PTA, February/March 2023) (BioSkryb, Durham, NC). Successfully amplified samples underwent library preparation and next-generation sequencing (NGS). Sequencing data were analyzed using the artificial intelligence algorithm PGTai^SM^ 2.0 (CooperSurgical, Inc.), which interprets sequencing data with an algorithm stack. The PGTai^SM^ 2.0 pipeline calls any region that statistically deviates from the baseline reference population and flags regions which are automatically classified as follows: < 20% euploid, 20–40% and 41–80% as low- and high-level mosaic, respectively, and > 80% as aneuploid (6,7).

For preimplantation genetic testing for monogenic disorders, the amplified trophectoderm biopsy samples were processed alongside genomic DNA isolated from maternal, paternal, and reference buccal swabs. From November 2019 to June 2022, all samples underwent karyomapping by microarray per the manufacturer’s instructions (Infinium Karyomapping Assay, Illumina), and data were analyzed with commercial software (BlueFuse, Illumina). From July 2022 until March 2023, cases were processed using a targeted NGS approach, and data were analyzed using proprietary software (PGT-M by NGS, CooperSurgical, Inc.). Both methods leverage genome-wide single nucleotide polymorphisms (SNPs) to determine haplotype inheritance based on genetic linkage, and reference(s) are used to determine the mutational status of each embryo for the gene or region of interest. When possible, causal genes were assessed through direct Sanger sequencing as well.

A usable embryo was defined by considering chromosomal status (euploid versus mosaic/aneuploid) and monogenic status (affected, unaffected, or carrier). Euploidy was defined as euploid or 20–40% mosaicism [9]. In addition, the results of PGT-M being compatible with transfer depended on the inheritance pattern for the gene tested and, in some cases, the sex of the embryo. Usable embryos included (1) completely unaffected embryos, (2) unaffected carriers of an AR disorder, (3) embryos not affected with an AD condition, and (4) female embryo carriers of an XLR condition. All carriers for an AR condition or female carriers of an XLR condition were considered usable. PGT-M may be testing for two to four variants, for example, in the gene CYP21A2 each partner has a different variant and in HBB each partner may also have a different variant so the embryo would be tested for four variants in two genes. In our study, unusable embryos were defined as mosaic embryos (40–80% mosaic) or aneuploid embryos (> 80% mosaic), along with embryos affected by a single-gene disorder.

Descriptive statistics were calculated for patient demographics and cycle characteristics, with continuous variables presented as means with standard deviations and categorical variables presented as frequencies and percentages. The one-gene and two-gene groups were compared using the Kruskal–Wallis test for continuous variables and the chi-square test for categorical variables. Analysis was conducted on a per-cycle basis rather than a per-patient basis. Statistical significance was defined as a *p*-value < 0.05, and all analyses were performed using SAS 9.4 (SAS Institute, Cary, NC).

## Results

### Demographics and descriptive data

A total of 4634 IVF cycles with PGT-A and PGT-M were included in the study. Among these, 146 cycles (3.15%) used PGT-M for detecting variants in two genes and 4488 cycles (96.85%) for one gene (Table 1). Mean patient age was similar in both groups (34.4 years in both; *p* = NS). There was also no difference in percent of patients < 35 years of age, comprising 55.9% of cycles in the one-gene group and 54.8% in the two-gene group (*p* = NS). Patients testing for variants in two genes underwent a significantly higher mean number of cycles compared to those testing for variants in one gene (1.81 vs 1.55 cycles; *p* = 0.007). Additionally, the mean number of embryos biopsied per cycle was significantly higher in the two-gene group for all ages (5.3 vs 4.6 embryos; *p* = 0.027). Specifically, this difference remained significant in patients younger than 35 years (6.15 vs 5.09; *p* = 0.027). However, in older age groups, the number of embryos per cycle was comparable between the two groups.

**Table 1:** Cycle characteristics

	PGT-A + PGT-M for two genes	PGT-A + PGT-M for one gene	*p*-value
Total # of cycles	146	4488	
Total # of patients	80	2903	
Patient age, mean (SD)	34.4 (3.45)	34.4 (3.92)	0.934^a^
Patient age, % (*n*)			0.376^b^
< 35	54.8 (80)	55.9 (2510)	
35–37	19.9 (29)	18.0 (808)	
38–40	20.5 (30)	18.4 (826)	
41–42	4.8 (7)	5.3 (236)	
43–45	-	2.4 (108)	
# of embryos per cycle, mean (SD)			0.027^a^
< *35*	6.15 (4.22)	5.09 (3.91)	
*35–37*	3.72 (2.09)	4.38 (3.42)	
*38–40*	4.17 (2.48)	3.83 (2.95)	
*41–42*	7.14 (3.80)	3.57 (2.83)	
> 42	-	3.10 (2.37)	
All age groups	5.31 (3.70)	4.60 (3.63)	
#Cycles per patient, mean (SD)			0.007^a^
< 35	1.70 (1.08)	1.47 (0.84)	
35–37	2.07 (0.92)	1.57 (0.87)	
38–40	1.81 (1.11)	1.73 (1.17)	
41–42	2.33 (2.31)	1.78 (1.10)	
> 42	-	1.55 (0.95)	
All age groups	1.81 (1.10)	1.55 (0.93)	

^a^Kruskal-Wallis *p*-value

^b^Chi-square *p*-value

The most commonly tested variant when PGT-M was performed for a single gene was FMR1 (10.23% of cycles) followed by CFTR (7.71%; Table 2). Other frequently tested variants in genes included BRCA1 (4.21%), HTT (4.03%), and HBB (3.14%). When PGT-M was performed for two genes, CFTR and FMR1 were the most tested for combination, appearing in 14.38% and 13.70% of cycles, respectively. PKD1, BRCA1, and BRCA2 were also among the most frequently tested genes on a per cycle basis.

**Table 2:** Most common genes in IVF + PGT-M cycles performed for one and two genes

	PGT-M for two genes *N* = 146 (*n*, % of cycles)	PGT-M for one gene *N* = 4488 (*n*, % of cycles)
1	CFTR (21, 14.4%)	FMR1 (459, 10.2%)
2	FMR1 (20, 13.7%)	CFTR (346, 7.7%)
3	PDK1 (14, 9.6%)	BRCA1 (189, 4.2%)
4	BRCA1 (13, 8.9%)	HTT (181, 4.0%)
5	BRCA2 (9, 6.2%)	HBB (141, 3.1%)

Gene, full gene name, and inheritance patterns: *CFTR*, Cystic Fibrosis Transmembrane Conductance Regulator (autosomal recessive); *FMR1*, Fragile X Messenger Ribonucleoprotein 1 (X-linked dominant); *PDK1*, Pyruvate Dehydrogenase Kinase, Isozyme 1 (autosomal recessive); *BRCA1*, Breast Cancer 1, Early Onset (autosomal dominant); *BRCA2*, Breast Cancer 2, Early Onset (autosomal dominant); *HTT*, Huntingtin (autosomal dominant); *HBB*, Hemoglobin Subunit Beta (autosomal recessive)

When looking at the most frequent inheritance pattern combination among patients testing for variants in two genes, AD + AD was the most common (35.62%), followed by AD + AR (25.34%) and AR + AR (17.12%; Table 3). X-linked conditions were tested for the least frequently, with XL + AR making up 16.44%, AD + X-linked 4.80% of cycles, and X-linked + X-linked comprising 0.68% of cycles.

**Table 3:** Percent of PGT-M for two-gene cycles by combination of inheritance patterns

Inheritance pattern combination	PGT-M cycles with two genes *N* = 146 % (*n*)	Unique patients	Cycles per patient Mean (SD)
AR + AR	17.12 (25)	16	1.6 (1.1)
AR + AD	25.34 (37)	19	1.9 (1.1)
AR + X linked	16.44 (24)	16	1.5 (0.8)
AD + AD	35.62 (52)	25	2.1 (1.3)
AD + X linked	4.80 (7)	5	1.4 (0.5)
X linked + X linked	0.68 (1)	1	1 (-)

*AR*, autosomal recessive; *AD*, autosomal dominant

### Likelihood of obtaining a usable embryo for transfer

In the unadjusted analysis, the likelihood of obtaining at least one usable embryo per cycle was significantly lower in patients aged 35–37 who tested for variants in two genes compared to those testing for one gene (0.552 vs 0.744; *p* = 0.021). Among patients younger than 35 years, there was a similar trend toward a lower likelihood in the two-gene group (0.75 vs 0.83), although this difference did not reach statistical significance (*p* = 0.082). No differences were observed among patients aged 38 years or older. However, after adjusting for the number of embryos biopsied, patients aged < 35 years testing for variants in two genes had significantly lower odds of obtaining a usable embryo compared to those testing for one gene (adjusted OR 0.41, 95% CI 0.22–0.76; *p* = 0.004). This association remained significant in the 35–37 age group (adjusted OR 0.40, 95% CI 0.18–0.89; *p* = 0.025). No significant difference is observed in patients aged 38–40 years or 41–42 years, though the older age groups are limited by small sample size (Table 4).

**Table 4:** Likelihood of having at least one usable embryo per cycle with PGT-A + PGT-M cycles testing one and two genes, stratified by age

	PGT-M for two genes *N* = 146 % (SE)	PGT-M for one gene *N* = 4488 % (SE)	Unadjusted *p*-value^a^	Adjusted OR (95% CI)^b^	Adjusted *p*-value^b^
< 35	0.750 (0.048)	0.825 (0.008)	0.083	0.41 (0.22–0.76)	0.004*
35–37	0.552 (0.092)	0.744 (0.015)	0.021	0.40 (0.18–0.89)	0.025*
38–40	0.667 (0.086)	0.657 (0.017)	0.916	0.79 (0.33–1.86)	0.586
41–42	0.429 (0.187)	0.517 (0.033)	0.645	0.25 (0.04–1.39)	0.113
> 42	-	0.278 (0.043)	-	-	-

^a^Chi-square test

^b^Adjusted for the number of embryos biopsied using logistic regression, with PGT-M for one gene as the reference group

^*^p < 0.05

An additional analysis was done to assess the number of cycles that were lost to PGT-A in those doing PGT-M for two variants, demonstrating the percent of cycles with at least one usable embryo dropped from 84.9 to 67.8% with the addition of PGT-A (Supplementary Table 1).

## Discussion

In this study, we found that PGT-M testing for variants in two genes compared with in one gene, in addition to PGT-A, is associated with a lower likelihood of obtaining at least one usable embryo per cycle in younger age groups, while no statistically significant difference was observed in older groups. Use of PGT-M provides the opportunity to select embryos for transfer that are not affected by known inheritable conditions. With an increasing number of patients with known genes from increased use of ECS panels and increased genetic testing for hereditary cancer syndromes, patients are more likely to encounter situations where they carry genes for more than one pathologic condition [3]. PGT-M for simultaneous screening of embryos for variants in two specific genes, in addition to PGT-A, expands diagnostic capabilities but may limit the likelihood of having a usable, unaffected euploid embryo. The mode of inheritance of the gene and whether carrier status is acceptable for transfer are important considerations impacting outcomes. Understanding the likelihood of having an embryo for transfer in IVF cycles using PGT-M for variants in two genes is essential for providers to set realistic expectations for patients prior to treatment initiation. The primary outcome of this study was to determine the chance of having at least one euploid, unaffected embryo per cycle among patients who undergo IVF with PGT-M for variants in one versus two genes, stratified by patient age, in addition to offering insight into specifics of PGT-M utilization for two genes.

Prior literature has looked almost exclusively at embryo and IVF outcomes for cycles undergoing PGT-M for variant(s) in a single gene compared to no testing or PGT-A alone. While all cycles in our study used both PGT-M and PGT-A, a subanalysis found an 11.0% decrease in cycles with at least one usable embryo with the addition of PGT-A. There was no consistent pattern in the drop-out by age group, possibly related to small sample size, variable number of embryos per biopsy in different age groups, as well as focus on at least one usable embryo per cycle, not number of usable embryos. Our group previously analyzed 72,522 IVF cycles between 2019 and 2023, including 4255 cycles with combined PGT-M + PGT-A and 68,267 with PGT-A only, focusing on the likelihood of obtaining at least one embryo that is both genetically unaffected and chromosomally euploid. No significant difference was found in age-specific aneuploidy rates between groups, but cycles with concurrent PGT-M were overall less likely to yield a usable embryo in patients aged ≤ 40 years, except in selected cohorts (AR genes in patients 38–40, XL genes in patients 35–37) [8]. Similarly, another retrospective analysis of 181 PGT-M cycles demonstrated that the likelihood of obtaining embryos suitable for transfer significantly decreased when PGT-M was added to PGT-A [10].

While not the primary focus of our current study, several other studies have looked at the utility of adding PGT-A to PGT-M cycles testing for variant(s) in one gene. In a retrospective PGT-M cycle analysis of 163 embryos, researchers found that although 55.7% of blastocysts were unaffected with a single-gene disorder, only 27.5% were both unaffected and euploid [11]. In a single-center cohort study involving 355 embryos, 54.7% of embryos were considered usable for transfer after PGT-M criteria alone; however, only 25.6% were when both PGT-M and PGT-A were applied. While usable embryos decreased, the addition of PGT-A improved per-transfer outcomes by increasing rates of implantation and lowering rates of miscarriage [12]. Another small, retrospective chart review of 181 IVF cycles found that cycles with PGT-M for AR genes had a modest reduction in usable embryos per cycle when PGT-A was added (mean embryos 2.66 vs 2.88, *p* = NS), but the impact was substantially greater for autosomal dominant and X-linked indications [13]. Similarly, a cohort undergoing PGT-M for AD conditions demonstrated that although 94% of cycles yielded at least one genetically unaffected embryo, only 69.9% yielded an embryo that was both unaffected and euploid [14]. Transferable embryo proportion dropped from ~ 49.3% with PGT-M alone to ~ 39.1% when PGT-A was added [14].

Taken together, these studies show that PGT-M for variant(s) in a single gene reduces the final pool of transferable embryos, particularly in younger patients and those testing for AD or XL genes where unaffected embryos are proportionately fewer. This is not surprisingly compounded by the addition of PGT-A. Notably, most of these studies focused on usable embryos and not clinical outcomes. While selecting only euploid, genetically unaffected embryos improves implantation and reduces miscarriage per transfer [11], it also increases the likelihood of no embryos to transfer [8, 10]. There is almost no prior literature on PGT-M that specifically focuses on testing variants in two genes. To our knowledge, the only prior study looking specifically at PGT-M for variants in two genes was a small case series limited by a small sample size of 23 patients at a single center [7].

In our study, a total of 4634 IVF cycles utilizing PGT-M were analyzed, of which 146 cycles (3.15%) used PGT-M for variants in two genes. This is the largest study to-date of PGT-M for two genes and includes a large comparison group of patients undergoing PGT-M for one gene. After adjusting for the number of embryos biopsied, patients aged < 35 years testing for two genes had significantly lower odds of obtaining a usable embryo compared to those testing for one gene (adjusted OR 0.41, 95% CI 0.22–0.76, *p* = 0.004). This association remained significant in the 35–37 age group (adjusted OR 0.40, 95% CI 0.18–0.89, *p* = 0.025). No significant difference was observed in patients aged 38–40 years or 41–42 years. We initially hypothesized there would be a lower usable embryo rate when PGT-M was utilized across age groups. The absence of a significant difference in the older age groups may reflect limited power due to small sample size, differences in inheritance patterns, underlying prognosis, or the number of embryos biopsied per cycle. These could also contribute to the age-stratified pattern. Additionally, in older cohorts, the stronger impact of advanced maternal age on aneuploidy may have overridden the influence of PGT-M on the usable embryo rate. The absence of statistical significance in the 38–40 group does not necessarily indicate absence of effect, and larger cohorts will be needed to determine whether these patterns reflect real biological differences or residual confounding.

Our study also looked at the frequency of inheritance pattern combinations. The most common combinations of inheritance patterns when two genes were tested were two AD genes (*n* = 52, 35%) followed by an AD and AR gene (*n* = 37, 25%) and two AR genes (*n* = 25, 17%). Our study also describes the most tested-for genes when PGT-M is performed for variants in two genes. While the cohort size was too small to control specific inheritance pattern combinations in our usable embryo analysis, our study is the first to further detail and describe this unique and growing cohort of patients.

A primary strength of this study is its large sample size. Use of PGT-M for variants in two genes is rare and our dataset from a large genetics laboratory allowed for sufficient numbers for analysis of this cohort. Additionally, IVF stimulation cycles were performed at multiple centers from across the country and not a single site, allowing for increased generalizability. The study does have some notable limitations, including the retrospective design which introduces potential for selection bias among patients with access to IVF and utilization of PGT-M. Additionally, while the data was from multiple fertility centers, all genetic testing was run at a single genetics laboratory which may introduce laboratory-specific procedural bias. Analysis was conducted on a per cycle basis rather than a per patient basis, meaning that the potential inclusion of multiple cycles from the same patient is not an independent observation. Further studies would be strengthened by looking at a similar question on a per patient basis rather than a per cycle basis, as well as linking these genetic testing results with clinical outcomes including ongoing pregnancy and live birth.

## Conclusion

Quantifying the likelihood of obtaining a usable embryo when doing PGT-A with PGT-M for variants in two genes by patient age is a helpful tool when counseling patients who are deciding whether to test for two genes. After adjusting for the number of embryos biopsied, there were significantly lower odds of obtaining a usable embryo in patients who tested for variants in two vs one gene among those aged < 35 years and 35–37 years. No statistically significant difference was seen among patients 38–40 years or 41–42 years in the odds of obtaining at least one usable embryo per cycle; however, this null result should be interpreted with caution given the limited sample size and the high baseline aneuploidy rates. This study reinforces the significant impact of oocyte age on aneuploidy and should be included when counseling patients who present for PGT-M. The decision to test for variants in two genes can be a difficult one, and this study provides key information to inform counseling for patients faced with this situation during reproductive planning.

## Supplementary Information

Below is the link to the electronic supplementary material.

Supplementary file1 (DOCX 14 KB)

## Data Availability

Data for analysis provided by preimplantation genetic testing laboratory.
