# Supplementary material for: Likelihood of obtaining a usable embryo for transfer after IVF with PGT-A and PGT-M for variants in two genes
Source: J Assist Reprod Genet. 2026 Apr 24;43(6):1749–55. doi: 10.1007/s10815-026-03868-4 (PMC13319311; doi:10.1007/s10815-026-03868-4)
Supplement: Supplementary file 1 — Supplementary file1 (DOCX 14 KB) [file 10815_2026_3868_MOESM1_ESM.docx]

**Supplemental Table 1. Number of cycles with at least one usable embryo per cycle in cycles using PGT-M for two variants, with and without PGT-A results**



| **SART Age Group** | **Total Cycles** | **1+ Embryo Usable by PGT-M Alone, n (%)** | **1+ Embryo Usable after PGT-M and PGT-A, n (%)** | **Lost to PGT-A, n (%)** |
| --- | --- | --- | --- | --- |
| <35 | 80 | 65 (81.3) | 60 (75.0) | 5 (7.7) |
| 35-37 | 29 | 23 (79.3) | 16 (55.2) | 7 (30.4) |
| 38-40 | 30 | 20 (66.7) | 20 (66.7) | 0 (0) |
| 41-42 | 7 | 7 (100.0) | 3 (42.9) | 4 (57.1) |
| >42 | **-** | **-** | **-** | **-** |
| Total | 146 | 115 (78.8) | 99 (67.8) | 16 (13.9) |
